# Supplementary material for: Compositional data analysis enables statistical rigor in comparative glycomics
Source: Nat Commun. 2025 Jan 18;16:795. doi: 10.1038/s41467-025-56249-3 (PMC11748655; doi:10.1038/s41467-025-56249-3)
Supplement: Supplementary file 1 — Supplementary Information [file 41467_2025_56249_MOESM1_ESM.pdf]

Compositional Data Analysis Enables Statistical Rigor in Comparative Glycomics

Alexander R. Bennett[1], Jon Lundstrøm[2,3], Sayantani Chatterjee[4], Morten Thaysen-Andersen[4,5], Daniel Bojar[2,3,*]

[1]Department of Medical Biochemistry, Institute of Biomedicine, University of Gothenburg, 41390 Gothenburg, Sweden.

[2]Department of Chemistry and Molecular Biology, University of Gothenburg, 41390 Gothenburg, Sweden.

[3]Wallenberg Centre for Molecular and Translational Medicine, University of Gothenburg, 41390 Gothenburg, Sweden.

[4]School of Natural Sciences, Faculty of Science and Engineering, Macquarie University, Sydney, Australia

[5]Institute for Glyco-core Research (iGCORE), Nagoya University, Nagoya, Japan.

[*]Corresponding author, e-mail: daniel.bojar@gu.se

Supplementary Figures

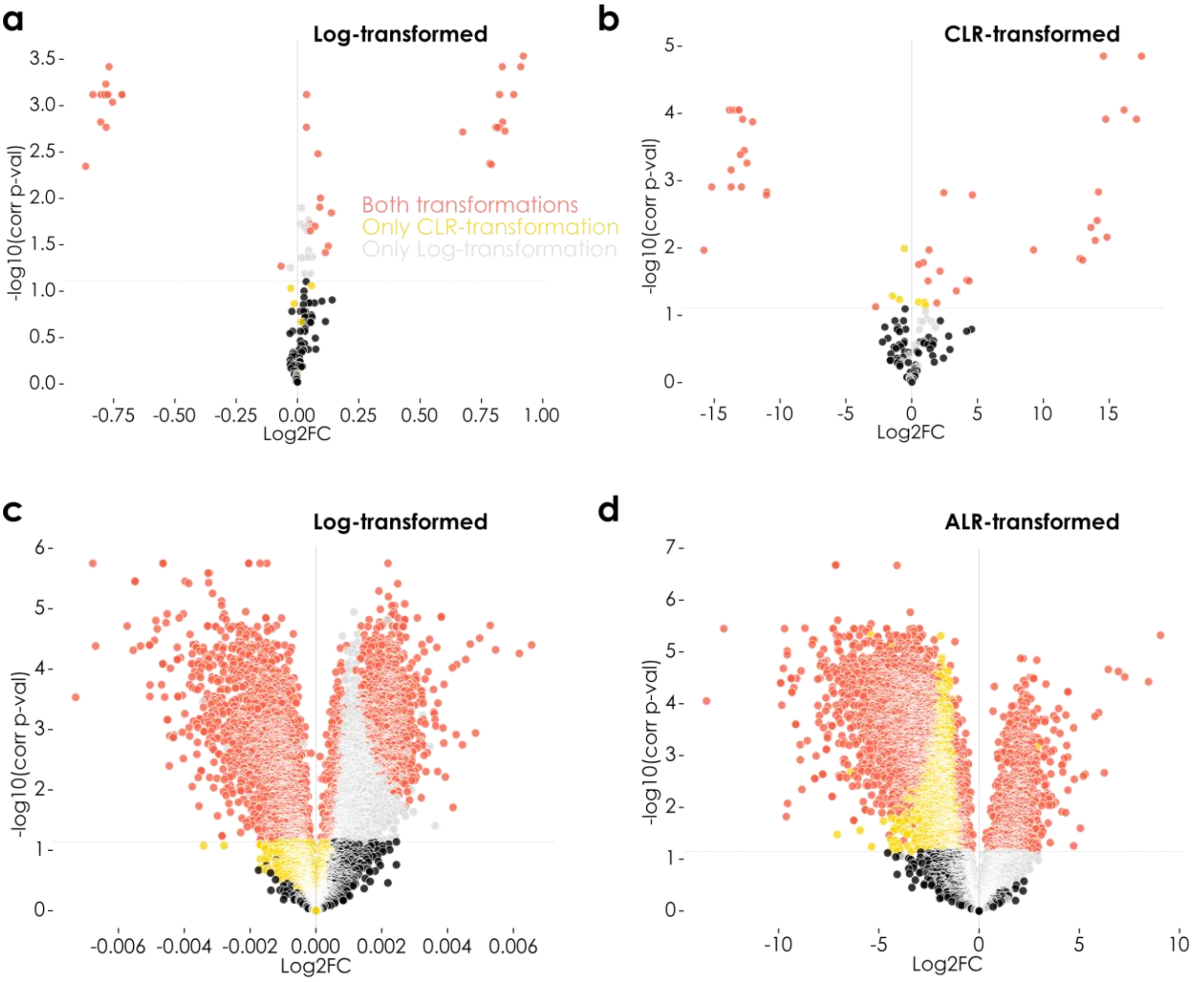

Supplementary Figure 1. Analyzing glycoproteomics data via CLR-transformation. a-d) Immunoglobulin glycoproteomics data from human milk (a-b, colostrum vs mature, n = 3 per condition, Wang et al., *J Agric Food Chem*, 2021) or two liver cancer cell lines (c-d, Hep3B vs 97L, n = 4 per cell line, Kong et al., *Nat Commun*, 2022) were analyzed with the *get_differential_expression* function in glycowork (version 1.3), with either log_2-transformed relative abundances (a, c) or ALR/CLR-transformed relative abundances (b, d). Shown are volcano plots, where glycopeptides are colored red if both transformations identified their differential expression, yellow if only CLR-transformations recovered their differential expression, and grey if only log_2-transformations identified differential expression. On average, glycopeptides with lower fold-changes were more likely to only be captured as significant by CLR-transformation. The horizontal line indicates the sample-size appropriate significance thresholds of alpha = 0.08 or 0.07, respectively. Source data are provided as a Source Data file.

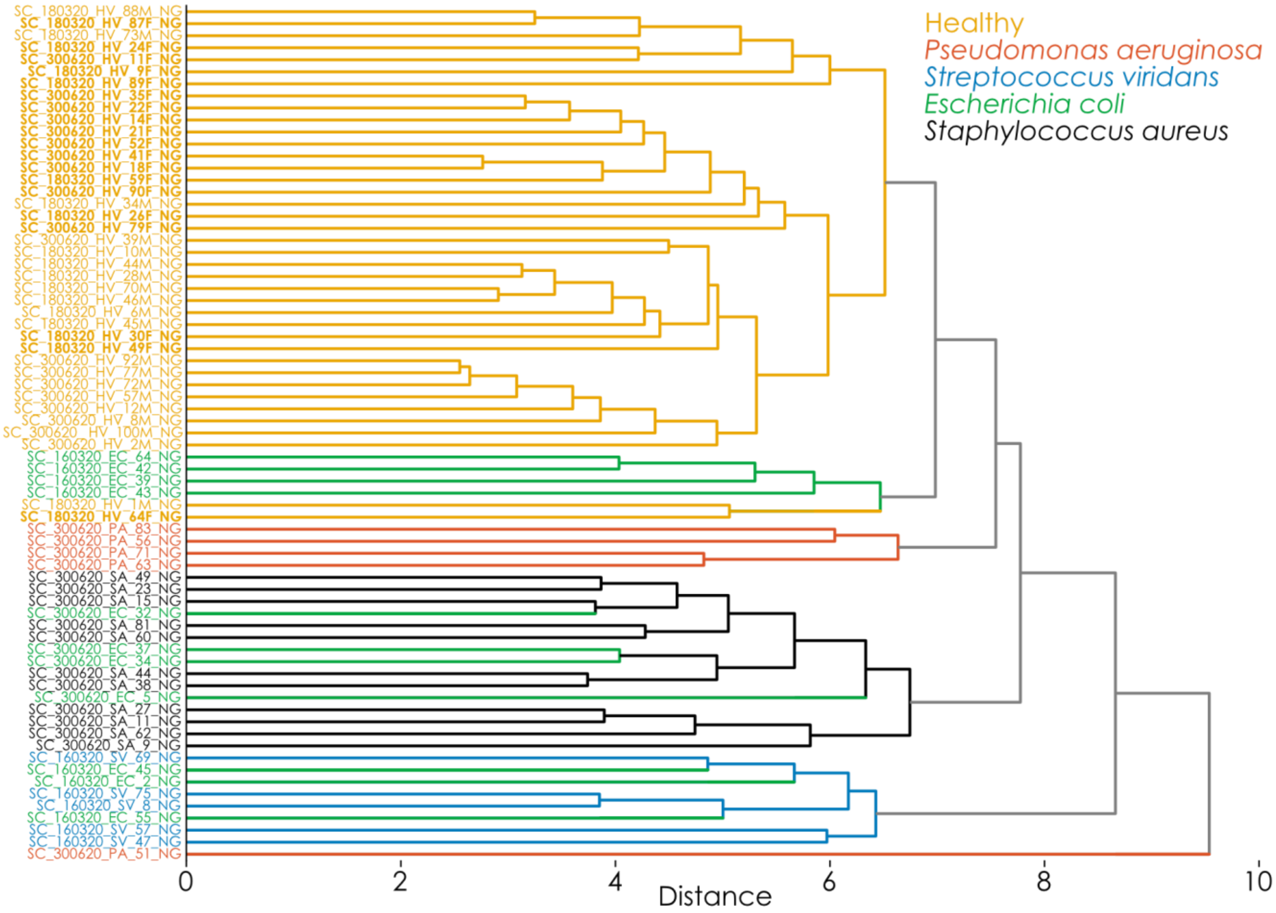

Supplementary Figure 2. ALR-transformed data yield superior clustering. Serum *N*-glycome data from healthy volunteers or bacteria-infected patients (total N = 71; Chatterjee et al., *J Clin Med*, 2021) were ALR-transformed via the *get_heatmap* function (glycowork, version 1.3). Then, an unsupervised hierarchical clustering was performed via the UPGMA algorithm on Euclidean distances. Patient classes are indicated by their color and, for healthy volunteers, female participants were indicated by bolding their ID, demonstrating even a clustering within the class by the known glyco-modifier sex.

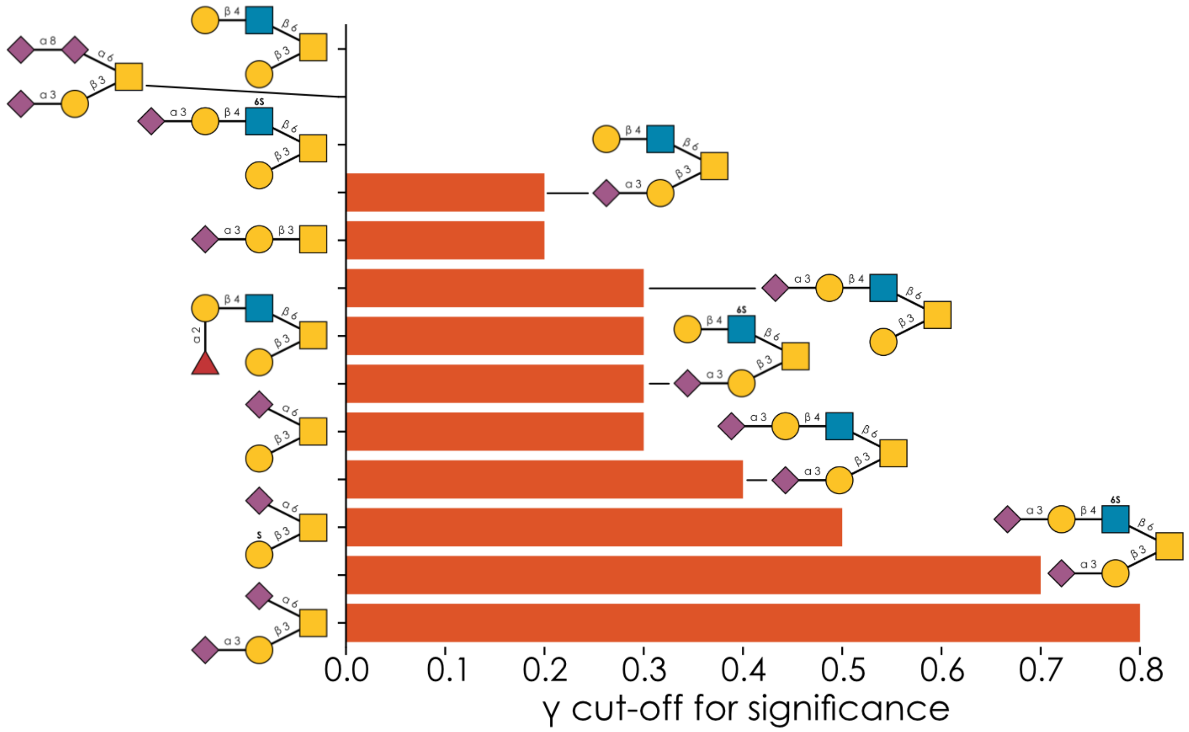

Supplementary Figure 3. Scale uncertainty as a measure for effect robustness. For the basal cell carcinoma *O*-glycomics dataset (Möginger et al., *Front Oncol*, 2018), we calculated the differential expression between paired healthy and cancerous tissue (N = 40) using the *get_differential_expression* function (glycowork, version 1.3) and different values of γ as scale uncertainty, ranging from 0 to 1. Recorded as a bar graph, for each structure, is the value of γ at which no significant difference could be established ($p < 0.044$). Greater values of this γ-cutoff then indicate more robust effects, as they persist even under great scale uncertainty. Glycans are depicted via the Symbol Nomenclature for Glycans (SNFG). Source data are provided as a Source Data file.

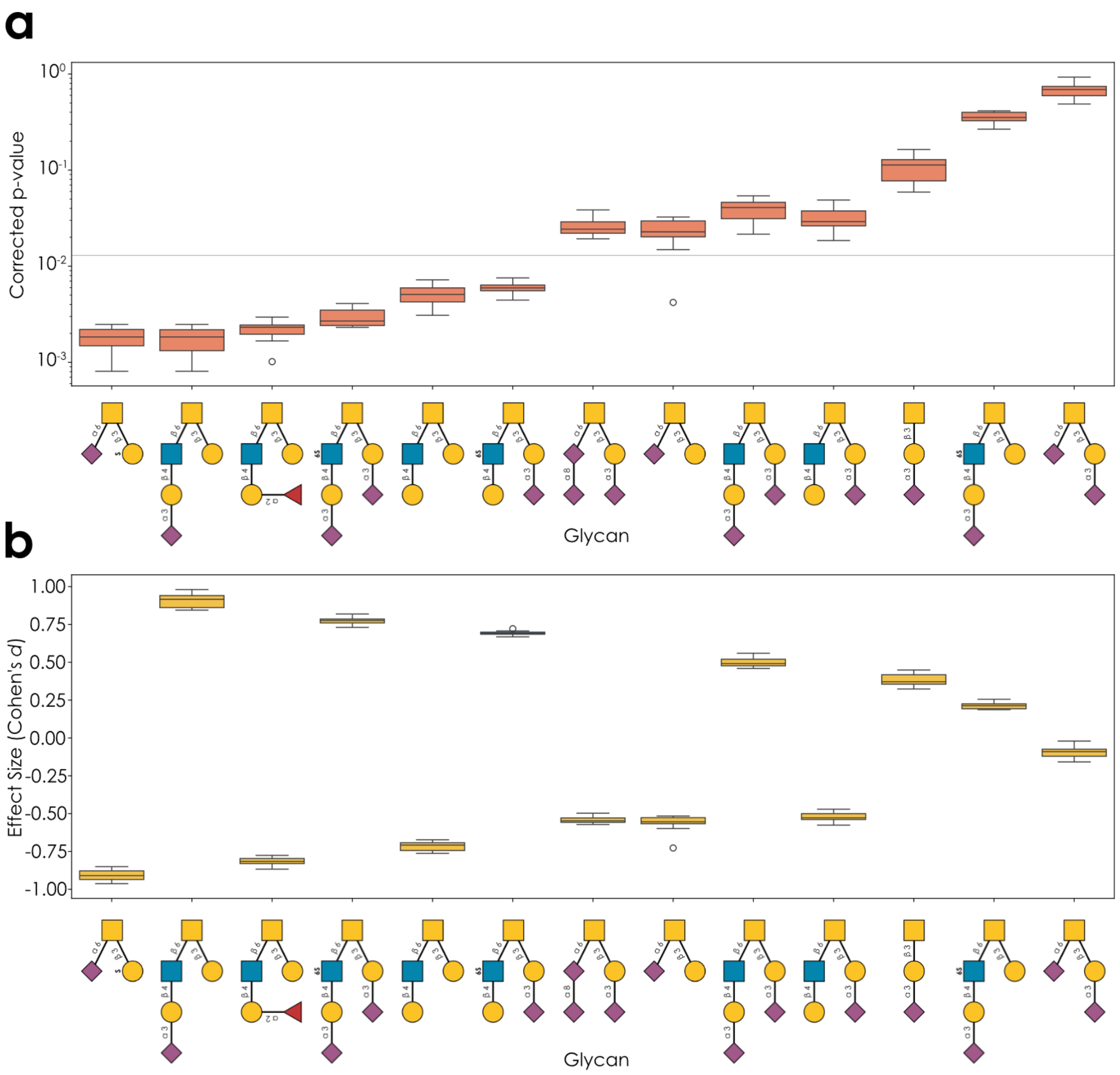

Supplementary Figure 4. Variance of default γ values does not impact reproducibility of analyses. a-b) For the basal cell carcinoma *O*-glycomics dataset mentioned in Supplementary Fig. 3 (N = 40), we reran *get_differential_expression* with the same parameters yet without fixing the random seed. Since scale uncertainty will lead to randomly drawing variance from a distribution, both p-values (corrected for multiple testing) and effect sizes will vary between runs, if the random seed is not fixed. Yet we report that both qualitatively (a; i.e., which glycans are statistically significant in their differential abundance) and quantitatively (b; i.e., the size of the observed effect), our analyses at the default values of γ remain reproducible, even in the absence of a fixed seed. A horizontal line in panel a indicates the cut-off for statistical significance (p = 0.044 at a sample size of N = 40). Data are depicted as mean values, with box edges indicating quartiles, whiskers indicating the remaining data distribution up to the 95% confidence interval, and circles indicate outliers, outside the 95% CI. Source data are provided as a Source Data file.

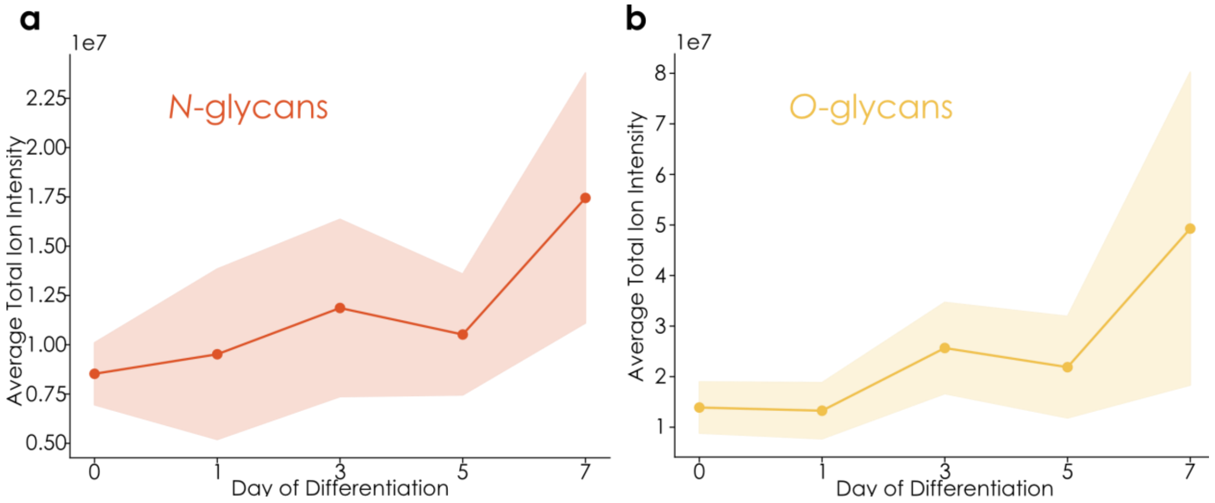

Supplementary Figure 5. Change in total glycan signal over macrophage differentiation. a-b) For each day of macrophage differentiation (N = 42; Hinneburg et al., *Glycobiology*, 2020), for both *N*-glycans (a) and *O*-glycans (b), the integrated ion intensity of all glycans was summed for each sample and averaged across donors and replicates. Shown are the mean total ion intensity as a line plot, together with a 95% confidence band. Source data are provided as a Source Data file.

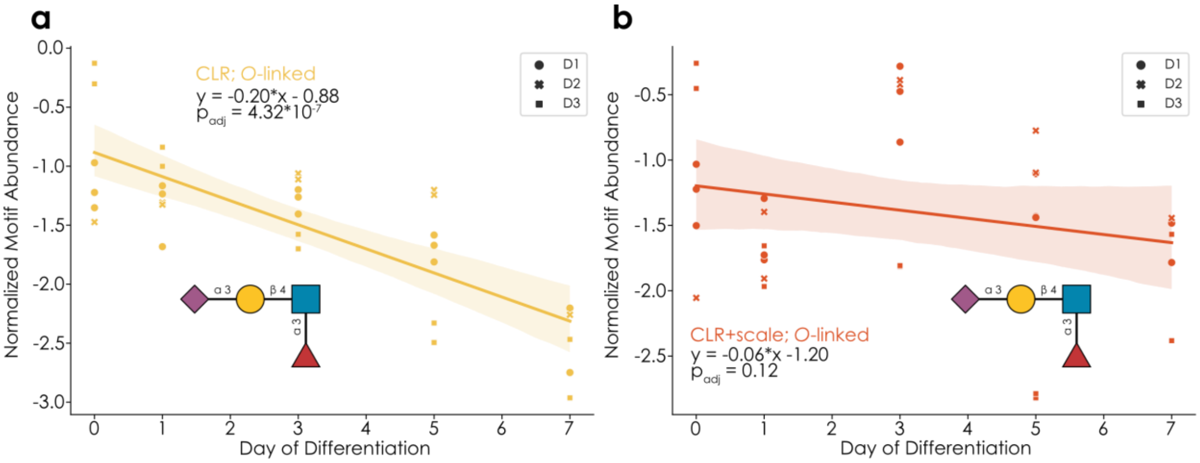

Supplementary Figure 6. Relative and absolute decrease of sialyl-Lewis X in *O*-glycans during macrophage differentiation. a-b) CLR-transformed *O*-glycomics data from a longitudinal macrophage differentiation dataset (N = 42; Hinneburg et al., *Glycobiology*, 2020) was analyzed similar to Figure 3b-g, analyzing the temporal behavior of sialyl-Lewis X expression with a scale uncertainty model (a) or an informed scale model (b). Source data are provided as a Source Data file.

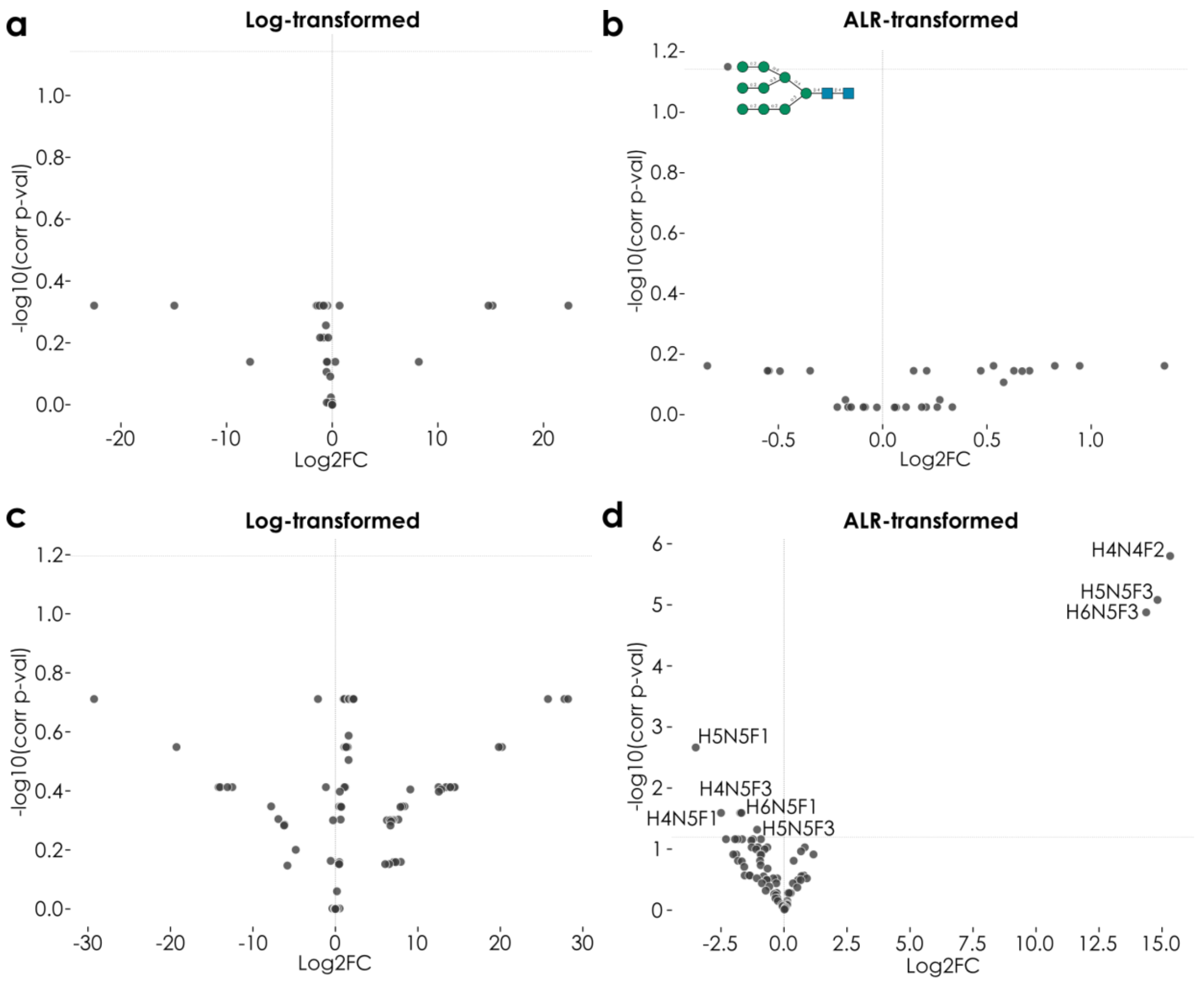

Supplementary Figure 7. Directly using glycomics ion intensities for analyses suffers from low sensitivity. a-d) *N*-glycomics data from a chronic lymphocytic leukemia (a-b; n = 8) and a colorectal carcinoma dataset (c-d; n = 12) from Chatterjee et al., *Oncotarget*, 2021, were used for differential expression analyses, using either log-transformed ion intensities (a, c) or ALR-transformed relative abundances (b, d). Shown are volcano plots, where a horizontal line indicates the alpha-cutoff for significant differential expression. Differentially expressed glycans are annotated with their SNFG representation or their composition if no structure was available. Source data are provided as a Source Data file.

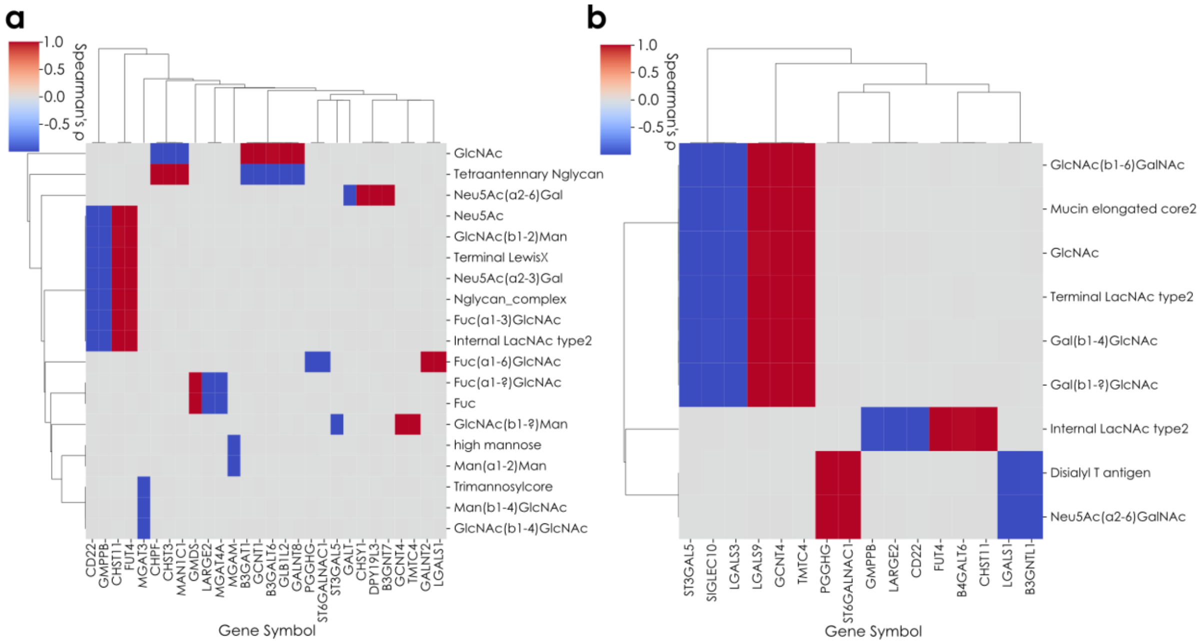

Supplementary Figure 8. Cross-correlation between glycan motifs and transcriptomics using compositional data analysis. a-b) Using B-cell glycomics and transcriptomics data from Oliveira et al., *Theranostics*, 2021, we filtered the list of differentially expressed for glycogenes, transformed both glycomics and transcriptomics data with CLR with a scale uncertainty model and calculated the cross-correlations between transcripts and *N*- (a) or *O*-glycan motifs (b). Shown are only significant correlations ($p < 0.083$, at $n = 5$), with their Spearman's rho as retrieved from the *get_SparCC* function (glycowork, version 1.3).
