# Supplementary material for: Compositional data analysis enables statistical rigor in comparative glycomics
Source: Nat Commun. 2025 Jan 18;16:795. doi: 10.1038/s41467-025-56249-3 (PMC11748655; doi:10.1038/s41467-025-56249-3)
Supplement: Supplementary file 2 — Description of Additional Supplementary Files [file 41467_2025_56249_MOESM2_ESM.pdf]

Description of Additional Supplementary Files

File Name: Supplementary Data 1

Description: For five glycan standards added to PGM, we measured their abundance via LC-ESI-MS. Three standards (m/z 893, 941, 1184) were always present in 2pmol/ul, whereas two standards (m/z 1010, 1111) were added in 2, 6, 12, and 18 pmol/ul in triplicates. Shown are the absolute ion intensities, relative abundances, and ALR-transformed abundances, using a glycan from PGM as the reference glycan.

File Name: Supplementary Data 2

Description: Time series analysis of O-glycan sequences during macrophage differentiation after CLR-transformation. P-values derive from two-tailed F-tests, with subsequent Benjamini-Hochberg correction for multiple testing.

File Name: Supplementary Data 3

Description: Time series analysis of O-glycan sequences during macrophage differentiation after CLR-transformation and using an informed scale model. P-values derive from two-tailed F-tests, with subsequent Benjamini-Hochberg correction for multiple testing.

File Name: Supplementary Data 4

Description: Time series analysis of O-glycan motifs during macrophage differentiation after CLR-transformation. P-values derive from two-tailed F-tests, with subsequent Benjamini-Hochberg correction for multiple testing.

File Name: Supplementary Data 5

Description: Time series analysis of O-glycan motifs during macrophage differentiation after CLR-transformation using an informed scale model. P-values derive from two-tailed F-tests, with subsequent Benjamini-Hochberg correction for multiple testing.

File Name: Supplementary Data 6

Description: Time series analysis of N-glycan sequences during macrophage differentiation after ALR-transformation. P-values derive from two-tailed F-tests, with subsequent Benjamini-Hochberg correction for multiple testing.

File Name: Supplementary Data 7

Description: Time series analysis of N-glycan sequences during macrophage differentiation after ALR-transformation and using an informed scale model. P-values derive from two-tailed F-tests, with subsequent Benjamini-Hochberg correction for multiple testing.

File Name: Supplementary Data 8

Description: Time series analysis of N-glycan motifs during macrophage differentiation after ALR-transformation. P-values derive from two-tailed F-tests, with subsequent Benjamini-Hochberg correction for multiple testing.

File Name: Supplementary Data 9

Description: Time series analysis of N-glycan motifs during macrophage differentiation after ALR-transformation using an informed scale model. P-values derive from two-tailed F-tests, with subsequent Benjamini-Hochberg correction for multiple testing.

File Name: Supplementary Data 10

Description: Differential expression of N-glycan motifs between non-transfected and mock-transfected HEK293 cells after CLR transformation. P-values derive from two-tailed Welch's t-test, followed by a Benjamini-Hochberg correction for multiple testing.

File Name: Supplementary Data 11

Description: Pan-cancer meta-analysis of pauci- and oligo-mannose N-glycans after CLR-transformation. P-values derive from two-tailed t-tests of the combined effect size, followed by a Benjamini-Hochberg correction for multiple testing.

File Name: Supplementary Data 12

Description: Pan-cancer meta-analysis of pauci- and oligo-mannose N-glycans after CLR-transformation with informed scale model. P-values derive from two-tailed t-tests of the combined effect size, followed by a Benjamini-Hochberg correction for multiple testing.

File Name: Supplementary Data 13

Description: Pan-cancer meta-analysis of pauci- and oligo-mannose N-glycans after CLR-transformation with informed scale model. P-values derive from two-tailed t-tests of the combined effect size, followed by a Benjamini-Hochberg correction for multiple testing.

File Name: Supplementary Data 14

Description: Pan-cancer meta-analysis of pauci- and oligo-mannose N-glycan motifs after CLR-transformation with informed scale model. P-values derive from two-tailed t-tests of the combined effect size, followed by a Benjamini-Hochberg correction for multiple testing.
